# Analysis of Factors That May Affect the Effectiveness of Ketogenic Diet Treatment in Pediatric and Adolescent Patients

**DOI:** 10.3390/jcm11030606

**Published:** 2022-01-25

**Authors:** Anna Winczewska-Wiktor, Adam Sebastian Hirschfeld, Magdalena Badura-Stronka, Paulina Komasińska-Piotrowska, Barbara Steinborn

**Affiliations:** 1 Department of Developmental Neurology, Poznan University of Medical Sciences, 60-355 Poznan, Poland; komasinska.paulina@spsk2.pl (P.K.-P.); bstein@ump.edu.pl (B.S.); 2 Department of Medical Genetics, Poznan University of Medical Sciences, 60-355 Poznan, Poland; adamhirschfeldmd@gmail.com (A.S.H.); badurastronka@ump.edu.pl (M.B.-S.); 3 Centers for Medical Genetics GENESIS, 60-406 Poznan, Poland

**Keywords:** ketogenic, diet, epilepsy, genetic, child

## Abstract

Purpose. The aim was to find predictors for ketogenic diet (KD) treatment effectiveness. In addition, recognized factors influencing the efficacy of KD were analyzed based on the ILAE (International League Against Epilepsy) proposed Classification and Definition of the Epilepsy Syndromes. Methods. A sample of 42 patients treated with KD were analyzed. The effectiveness of KD was assessed according to the type of diet, the type of seizures, and the known (KE) or undetermined genetic etiology (UNKE). The group of KE consisted of patients with *CACNA1S*, *CHD2*, *DEPDC5*, *KIF1A*, *PIGN*, *SCN1A*, *SCN8A*, *SLC2A1*, *SYNGAP1* pathogenic variants. The usefulness of the new Classification and Definition of Epilepsy Syndromes proposed by the ILAE was evaluated. Results. KD therapy was effective in 69.05% of cases. No significant correlation was observed with the type of diet used. KE was related to greater effectiveness after KD treatment. KD treatment was most effective in the reduction of non-focal seizures. Considering the ILAE proposed classification, it was found that KD efficacy was higher in patients with simultaneous focal and tonic-clonic seizures compared to patients with only tonic-clonic or focal seizures. Conclusion. The occurrence of focal seizures does not determine the potential ineffectiveness of treatment with a ketogenic diet. A significant efficacy of ketogenic diet treatment was observed in the group of patients with focal and generalized seizures, as well as epileptic and developmental encephalopathies. The etiology of epileptic seizures plays a more significant role. The new classification will make it easier to select patients who can benefit from this form of treatment.

## 1. Introduction

The ketogenic diet (KD) is a high fat, low carbohydrate, and adequate protein (1 g/kg) diet that has been presented as a possible treatment method for epilepsy since the 1920s [1,2]. Thus, a century of clinical observations and decades of research studies have suggested the possibility of using the KD to render neuronal network functions resistant to high electrical activity [3,4]. Unfortunately, the ketogenic diet has been sidelined for a long time due to the widespread development and use of antiseizure drugs (ASDs). However, in the last 20 years, further clinical data confirming the capacity of the KD to treat refractory epilepsy changed this situation and has encouraged its wider recognition [5,6]. The KD is currently an accepted treatment method for drug-resistant epilepsy, after two unsuccessful trials with antiseizure drugs, appropriately selected for the type of seizures or their background and used in suitable doses [7]. Additionally, it is used in the treatment of type 1 glucose transporter deficiency syndrome (GLUT1DS) and pyruvate dehydrogenase complex deficiency (PDCD) [8,9].

Although dietary treatment methods for drug-resistant epileptic seizures go back to 500 BC, their exact mechanism of action is not yet fully understood [10]. The multidirectional effect of the KD is emphasized through increasing the concentration of ketone bodies and polyunsaturated fatty acids, regulating apoptosis processes, modifying inflammatory reactions, and influencing the gut microbiota. At the same time, the KD alters the concentration of neurotransmitters and has a positive effect on the energy state of the cell [9,11,12,13].

Dietary treatment with the use of the KD aims to change the energy source in the central nervous system and bring the cell metabolism into a state of ketosis. Initially, only the classic forms of the KD were used for this purpose, with a ratio of fats to proteins and carbohydrates of 4:1 or 3:1 [14]. However, other descriptions define it as a deficiency in carbohydrate intake—5–10% of total daily calories, or 20–50 g per day [15]. The classic diet is very restrictive and can induce several side effects; therefore, the initiation of classic KD therapy takes place in a hospital setting [7]. This automatically limits the potential group of beneficiaries. However, positive, and sometimes spectacular, treatment effects have prompted attempts to modify the KD to be better tolerated, easier to handle, and able to be used in an outpatient setting while maintaining effectiveness and safety. This search led to the introduction of several KD variants, e.g., the modified Atkins diet (MAD), low-glycemic index treatment (LGIT), and the medium-chain triglyceride diet (MCTD) [9,16,17,18,19,20].

Most studies assessing potential contraindications to the KD are of low quality, with often a small sample size and relatively short duration [21]. For this reason, it is currently not possible to indicate contraindications to the KD, and it is recommended that the patient’s health status be assessed each time. Nevertheless, the available studies suggest that the KD is relatively safe to use in the majority of patients [22,23,24].

Several studies have been conducted to evaluate the effectiveness of KD epilepsy treatment in the pediatric population [14,25,26,27,28,29]. However, these attempts have proved difficult due to different patient enrolment criteria into the studies, different duration or types of KD used. The effectiveness, defined as a ≥50% reduction in the initial number of epileptic seizures, ranges from 35–85%, depending on the report. There is still a search for factors that may be associated with a greater likelihood of a good response to this form of treatment and, therefore, better selection of the group of patients who may benefit from it. Some authors observed a better response to therapy in patients diagnosed with generalized epilepsy or in younger patients [9,30,31].

The groups for which the KD is increasingly used are neurodegenerative and neurodevelopmental disorders [32]. However, some studies showed that in refractory epilepsy, particular types of epileptic seizures (mainly partial) predict a lack of good response [33,34]. Unfortunately, this has led to a perception among some clinicians that the KD is ineffective in such cases without considering the broader context of the disease symptoms. As a result, some study protocols ultimately ruled out patients presenting only partial seizures [35].

Sometimes it is forgotten that specific disease entities with a general set of symptoms may have various origins. Hence, KD therapy may have different results. That is why this study aimed to compare the effectiveness of the KD for various neurodegenerative and neurodevelopmental disorders depending on the genetic cause, either known or still unknown, and to assess whether partial seizures are a factor that determines its effectiveness.

## 2. Materials and Methods

The effectiveness of KD therapy was analyzed retrospectively in a group of 42 pediatric and adolescent patients. All patients were hospitalized in the Department of Developmental Neurology. The typical diagnostic procedures applicable to patients with epilepsy had been performed on all patients qualifying for treatment with a ketogenic diet. The diagnostics included: an assessment of the epileptic seizures morphology; a neurological, pediatric, or internist examination, depending on the age of the patients; a psychological assessment; EEG examination performed in wakefulness and sleep with hyperventilation and photostimulation; Holter EEG; magnetic resonance neuroimaging of the head; diagnostics to exclude metabolic diseases (analysis of the profile of organic acids in the urine with the GC/MS method (gas chromatography coupled with mass spectrometry); an analysis of the profile of acylcarnitines (in a dry drop of blood by the MS tandem method); and a genetic consultation (on the basis of which further genetic tests were performed, i.e., karyotype, array CGH, NGS panel or WES). During the treatment and subsequent visits to the clinic, the following were evaluated: the number of seizures, the occurrence of side effects, EEG recordings in wakefulness and sleep with hyperventilation and photostimulation, ECG, abdominal ultrasound examinations, blood count, liver tests, electrolytes, selenium, and l-carnitine concentration, the concentration of ketone bodies, thyroid parameters, calcium-phosphate metabolism, calcium-creatinine index, the concentration of antiepileptic drugs in the blood, and pancreatic parameters. In addition, patients were examined neurologically, pediatrically, or internistically (depending on the age of the patients), psychologically, and physiotherapeutically. The above-mentioned tests were performed monthly during the first three months of treatment, then once every three months during the next nine months of treatment, and then once every six months afterward. Between clinic visits, the patients’ parents monitored the concentration of ketone bodies at home. In addition, once a year, the patients were consulted cardiologically and urologically and had a densitometric examination performed. The effectiveness of KD was assessed according to the type of diet, the type of seizures, and the known (KE) or undetermined genetic etiology (UNKE). KE patients constituted 47.62% (20/42) of the study group, while UNKE patients constituted 52.38% (22/42). None of the patients was treated with vagus nerve stimulation or eligible for surgical treatment. Detailed data of the cohort are presented in Table 1.

Among the known underlying causes of the observed symptoms, there were *CACNA1S*, *CHD2*, *DEPDC5*, *KIF1A*, *PIGN*, *SCN1A*, *SCN8A*, *SLC2A1*, *SYNGAP1* pathogenic variants, trisomy 21, and deletion 15q11-13. In the UNKE group, the following syndromes were diagnosed: Doose syndrome (DS), juvenile epilepsy with absence seizures (JAE), Angelman-like syndrome (AS-like), Lennox–Gastaut syndrome (LGS), Rett-like syndrome (RS–like), febrile infection-related epilepsy syndrome (FIRES), and epilepsy with focal seizures of unknown etiology (UNFE). The patients with AS-like presented clinical symptoms observed in Angelman syndrome, i.e., dysmorphic features, intellectual disability, atactic gait, epileptic seizures, lack of speech, cheerful disposition, sleep disorders, changes in EEG. However, it was impossible to determine the observed symptoms’ molecular or cytogenetic basis. Furthermore, the group of patients with RS-like included girls with clinical symptoms resembling RS, i.e., slowing down of the normal psychomotor development observed around six months of age, lack of speech and normal motor development, lack of functional use of hands with movement stereotypes, episodes of hyperventilation and respiratory retention, intellectual disability, and seizures. Again, however, no molecular basis of the observed symptoms was found. Attempts to establish the molecular cause of these syndromes (e.g., array CGH, WES analysis, methylation of the *UBE3A* locus) were ineffective. The statistical methods used to analyze the obtained data are described for each of the results.

## 3. Results

Complete resolution of epileptic seizures was observed in 28.6% (12/42) of all patients; 75.00% (9/12) were patients with KE. Considering only the KE group of patients, 45.00% (9/20) achieved total seizure reduction. In the UNKE group, this percentage was lower—13.63% (3/22). Lack of KD treatment efficacy was defined as a reduction of ≤50%. This was the case in 10.00% (2/20) of patients in the KE group and 50.00% (11/22) in the UNKE group. Detailed data on the degree of seizure reduction in each group is presented in Table 2.

Key message: The ketogenic diet treatment was more effective in the group of patients with known etiology of epilepsy.

To check whether the known etiology of the disease was associated with greater effectiveness of seizure reduction after the KD treatment, Φ Yule’s correlation analysis was performed. It was found that the known etiology was related to the greater effectiveness of seizure reduction after dietary treatment (Φ = 0.37; *p* = 0.02).

The mean age of the patients in whom the diet was successful was 7.44 years (SD ± 5.75). In the group with an ineffective treatment, it was 5.04 years (SD ± 3.20). Among patients under two years of age (8/42), the KD proved successful in 62.50% (5/8) cases.

A point biserial correlation coefficient was performed to check whether the patients’ age impacted the effectiveness of seizure reduction using the KD. The result was statistically insignificant (*p* > 0.05). A Mann–Whitney U difference test was also performed to check for intergroup differences, the effect was statistically negligible (U = 135.00; *p* = 0.11).

Furthermore, we assessed whether the treatment duration affected the effectiveness of seizure reduction. A point-biserial correlation result was statistically insignificant.

**Figure 1:**
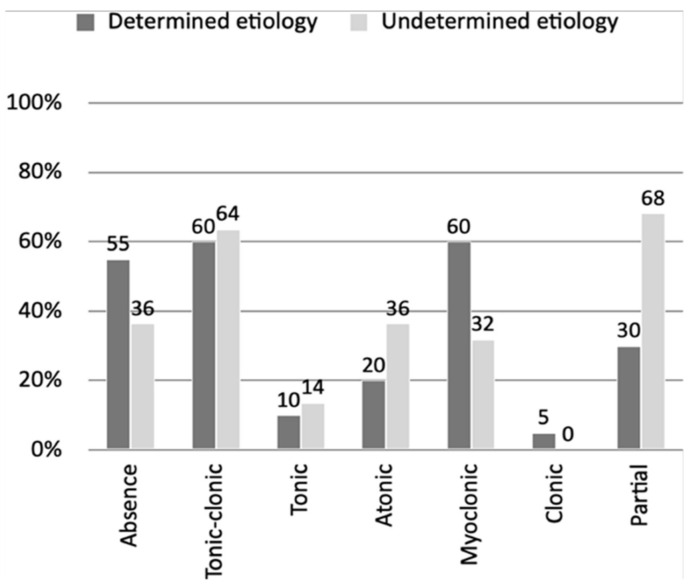
Percentage representation of the share of epileptic seizure types in groups with a defined and undefined genetic cause.

It was possible to obtain a detailed medical history in both groups and determine the types of epileptic seizures, as shown in Figure 1. Noteworthy was the high percentage of partial seizures in the UNKE group (68% vs. 30%).

After initiation of KD treatment, generalized tonic-clonic (2/12), atonic (2/8), and partial (3/6) seizures were found to be the most resistant in the KE group. On the other hand, the worst outcome in the UNKE group was seen for atonic (3/8), myoclonic (4/7), and partial (12/15) seizures.

Key message: Ketogenic diet treatment was most effective in the reduction of non-partial seizures.

Cramer’s V correlation analysis was performed to determine whether the type of symptoms influenced the efficacy of seizure reduction. It was found that the variables were related (V = 0.52; *p* = 0.01). To check whether there were differences in the effectiveness of the KD in the groups, the Kruskal–Wallis non-parametric equivalent of the one-way analysis of variance was calculated (due to the unequal size of the groups). The differences were statistically significant (chi^2^ = 11.24; *p* = 0.01). Thus, the most effective treatment was in the group with non-partial seizures, with moderate effectiveness with the mixed episode group, and least effectiveness for the partial seizures only group. The exact seizure reduction by type is presented in Table 3.

To determine whether the type of diet influenced the effectiveness of seizure reduction, Cramer’s V correlation analysis was performed, with no significant relationships between the variables found (V = 0.26; *p* = 0.90). A Mann–Whitney U test also showed no statistically significant differences in the type of diet and treatment effectiveness (U = 165.00; *p* = 0.42). Table 4 shows the size and effects for the analyzed diet therapy groups.

In some cases, patients discontinued the KD treatment (5/20 and 11/22, respectively, for KE and UNKE groups). The most common reason was the lack of expected effects and difficulty in maintaining the diet (80% vs. 91%). In eleven of these patients, treatment was completed within one year, and in the remaining five patients, treatment lasted more than one year. Among the UNKE patients who discontinued treatment, for two, the reason was complete resolution of seizures, and for the remaining nine, seizure reductions of below 50%. In the group of patients with KE, two patients had a reduction of seizures <50%. Despite a decrease in seizures >50% in the remaining three, the parents decided not to continue further therapy. In general, dietary treatment was well tolerated. No serious adverse events were observed. However, if the caregivers reported any side-effects, it was mainly at the beginning of the treatment (i.e., in the first month). The most commonly reported side effects were vomiting and reluctance to eat and drink. Among the symptoms persisting for more than one month after the KD initiation, which were the reason for discontinuation of therapy, weakness with apathy, and a feeling of hunger, were the most frequently mentioned. In addition, during therapy, patients reported somnolence (15% vs. 14%), nausea and vomiting (10% vs. 18%), excessive fatigue (10% vs. 4.5%), and increased symptoms of gastro-esophageal reflux (5% vs. 4.5%). Overall incidence of KD therapy side effects was 26.2% (11/42). None of the diets had an increased number of side effects compared to the others. However, the number of reported side effects was greater in the UNKE group 63.6% (7/11) compared to the KE group 36.4% (4/11).

Key message: Ketogenic diet treatment reduced the overall usage of anti-seizure drugs.

During the KD treatment in the KE group, in 40% (8/20) of cases, previously used medications were discontinued, while 25% (5/20) needed to start another ASD. At the same time, as many as 20% (4/20) of patients in this group used only the KD without any ASDs. In the UNKE patients group already using ASDs, these were discontinued in 36% of cases (8/22), while 55% of patients (12/22) had to take additional ASDs. None of the UNKE patients used the KD treatment without ASDs. More detailed data is presented in Table 5. To assess whether the effectiveness of the diet was related to the reduction of the number of ASDs used, Φ Yule’s correlation analysis was performed. It was found that higher efficacy was associated with decreased drug use (Φ = −0.34; *p* = 0.03).

We also asked parents about their views on the effect of the KD on improvement in cognitive functions. Their feelings were divided, as 50% (21/42) noticed progress, while the other half did not see positive effects. However, considering the parents’ responses concerning only a specific group, the improvement was reported in the KE group by 75% (15/20) and in the UNKE group by 27% (6/22).

## 4. Discussion

The KD is a recognized method of epilepsy treatment, although its exact mechanism of action remains elusive [36]. Interest in the dietary and surgical treatment of epilepsy has increased in recent years. This is because the percentage of drug-resistant patients remains stable despite the introduction of many new ASDs in the therapy process, side effects associated with these drugs, and restrictions on the use of specific ASDs in the youngest patients. With time, the range of indications for the use of the KD has expanded, and at the same time, less restrictive forms of treatment have been introduced. This paper analyzed factors that may be related to the potential effectiveness of the KD in a group of patients with drug-resistant epilepsy.

As mentioned, the KD is widely used in drug-resistant epilepsy as an add-on therapy after using two appropriately selected and dosed AEDs [7]. It is also the therapy of choice in patients with GLUT1DS and PDCD [37,38]. The potential mechanisms are by acting directly or indirectly on ion channels, increasing activation of seizure-reducing adenosine A1 receptors, enhancing mitochondrial function, or lowering of SIRT3-related oxidative stress [39].

Studies have indicated that KD therapy was effective in 35–85% of drug-resistant epilepsy patients [14,25,26,27,28]. Our results showed 69.05% (29/42) effectiveness in the whole cohort, demonstrating the importance of the KD in the treatment of refractory epilepsy.

Many different factors were analyzed to search for the optimal group to whom the KD treatment could be applied as most effective and safe. Some studies showed that a KD could be more effective in children under two years [31]. This may be due to the ineffectiveness of some ASDs on the maturing brain (lack of binding receptors), the multi-directional mechanism of action (including anti-inflammatory effects), and greater ease of dieting in young patients. At the same time, more and more data on efficacy in adult patients is emerging [40]. This finding is consistent with our results showing that KD treatment proved successful among patients under two years of age in 62.50% of cases (5/8). However, considering all patients’ data, we did not find a statistically significant correlation of age–effectiveness dependence. Thus, it seems that the etiology underlying the symptoms is more important than the age at which the treatment itself is started.

Another factor in the potential effectiveness of KD treatment is the time of therapy. Some studies observed a reduction in episodes by at least 50% in 75% of patients after three months of treatment, 73% after six months of treatment, and 90% after 12 months of treatment [41]. We found that the mean duration (years) of effective vs. ineffective KD treatment was 2.23 and 2.32. Further statistical investigation did not reveal any time–effectiveness correlation. However, reports suggest that a period of three months KD can be a predictive criterion of seizure remission over six months [42].

Apart from the mutations leading to GLUT1DS and PDCD, other mutations result in epileptic encephalopathies that respond well to KD treatment, such as *KCNQ2*, *SCN1A*, *SCN2A*, or *STXBP1* [43,44]. However, unfortunately, there are also those in which the response to the KD is much lower, e.g., *CDKL5* [9].

We found that in the KE group, complete resolution of seizures occurred in 75% of cases (9/12). Our results showed, apart from the apparent KD effectiveness in SLC2A1 patients (5/5), that all (3/3) *DEPDC5* and (1/1) *SYNGAP1* patients achieved a 100% seizure reduction. In particular, observation of an excellent response to the KD among patients with epilepsy caused by the *DEPDC5* mutation may be important. This is because of reported resistance to ASDs and the long-life persistence of seizures despite their use [45,46]. As to *SYNGAP1*-related epilepsy, there was an identified correlation between the genotype and pharmacoresistance [47]. Pathogenic variants in exons 4 or 5 were more responsive to ASDs than exons 8–15. Nevertheless, *SYNGAP1*-related epilepsy is refractory to classical ASDs in approximately 50% of cases [48]. Thus, the KD could be a possible treatment choice in such cases.

On the other hand, the KD treatment was unfortunately ineffective in 50% (1/2) of *SCN8A* and 100% (1/1) of *KIF1A* patients. The *KIF1A*-related spectrum is vast, and ASD-resistant epilepsy is common in some known phenotypes [49]. We did not find any reports of KD therapy attempts in the *KIF1A*-related spectrum with which to compare. As to *SCN8A*-related epilepsy, a recent study showed that KD therapy was effective in 55.56% (5/9) of patients [50]. Despite poor outcomes reported by other authors and us, there are some cases in which the KD could be of benefit for treatment.

At the same time, the syndromes with unknown genetic backgrounds, in which a significant improvement is observed after KD treatment, include DS, FIRES, and super-drug-resistant status epilepticus [51,52]. Indeed, we found that in the UNKE group, patients with a DS (2/5) and JAE (1/1) diagnosis achieved full seizure resolution. Furthermore, a favorable effect (>90% seizure reduction) was also obtained in AS-like (1/2), DS (1/5), LGS (1/3), and RS-like (1/3) patients. However, the KD treatment was ineffective in all (7/7) UNFE patients. Unfortunately, this was also the case in some of the DS (1/5), LGS (2/3), and RS-like (1/3) patients.

Overall, we found that the KD treatment was effective in 90% (18/20) of KE and 50% (11/22) of UNKE patients. Thus, it was more effective in patients with a known genetic underlying disease cause.

Regarding KD treatment effectiveness, a frequently raised issue is the type of seizures [33]. In agreement with available reports, we found that the partial seizure group had the worst response to treatment [34,53]. Firstly, both in the case of their co-occurrence and independent occurrence, they were the most challenging seizure type to be reduced—71.43% (15/21). Secondly, partial seizures were a statistically significant factor of worse response to the treatment. However, it should be taken into account that these data apply to all patients, and most partial seizures (15/21) occurred in the UNKE group. If only patients with known etiology are considered, partial seizures occurred in six cases, where the KD proved to be effective in 83.33% (5/6) of them.

Furthermore, partial seizures were the only seizure type in 9.52% (4/42) patients. In the case of the KE group, there was one patient with a complete reduction in seizures, and in the UNKE group, three patients with a 50% reduction, considered ineffective. Therefore, it seems that partial seizures should not discourage us from choosing the KD treatment, and we should be guided by knowledge of the underlying pathology. It should also be noted that a similar unfavorable correlation was seen with tonic-clonic seizures occurring as the only type. In most such cases (60%, 6/10), the KD was ineffective. Among other KD-resistant seizure types, atonic 41.66% (5/12), and myoclonic 25.32% (5/19) seizures were less often reported.

Due to side effects and the need to maintain a dietary regime, KD therapy is associated with the risk of withdrawal. In the group analyzed by us, the treatment was discontinued by 38.10% (16/42), while other studies have reported 33.5–35% [52,54]. In the UNKE group, the percentage of withdrawal was 50.0% and correlated precisely with the clinical assessment of KD ineffectiveness. However, in the KE group, it was 25.0% (5/20), while treatment was ineffective only in 10.0% of cases (2/20). This may indicate that the threshold for the effectiveness of KD therapy, being >50% reduction of seizures, adopted in many studies, does not meet parents’ expectations in some cases. In particular, the positive perception of the treatment effect may be influenced by other factors, such as the general improvement in cognitive function. According to the parents’ opinions, there was only a 75.0% improvement in cognitive function in the KE group. At the same time, there was 90.0% effectiveness in seizure reduction.

Our results also indicate that effective KD treatment was accompanied by a reduction in the ASDs used. In both the KE and UNKE groups, this percentage was similar, 40.0% vs. 36.4%. However, importantly, there was much less need to add another ASD in the KE group despite KD therapy (25.0% vs. 54.5%). The situation of possible drug reduction was also more favorable in the case of non-partial seizures (42.9%), where, in the case of partial seizures, it was 33.3%. It is worth noting that the KD does not affect the level of ASDs in the blood serum of children and adolescents [55,56]. Hence, the possibility of reducing the number of ASDs is closely related to the effectiveness of the KD. However, one report suggested a decreased efficacy of the KD among patients receiving lamotrigine [57]. In a group analyzed by us, only two patients received lamotrigine, and both achieved effective seizure reduction. Moreover, the Dravet syndrome cohort study showed that even in those without dramatic seizure reduction, there was an improvement in quality of life, and the number of ASDs was lowered to one or two [54].

Another aspect of the KD effectiveness is its type. Previous studies have not reported any correlation between the diet used and reducing a specific type of seizures [27,58,59]. We have also not found a superiority of a particular KD in terms of overall and type of seizure reduction. Therefore, it is likely that its general tolerance and clinical experience should determine the choice of a specific KD.

As for the tolerance of the KD, we did not find any diet to have significantly more or fewer side effects. Other authors have suggested that the MAD could have a lower number of side effects than the classical KD [60]. We did not observe such a correlation, and in terms of adverse effects, our data showed 21.4% (3/14) in the classical KD and 30.8% (4/13) in the MAD. Interestingly, we noticed that complaints were higher in the UNKE group than in the KE group, 63.6% vs. 36.4%. Probably responsible for this effect was the greater number of ASDs taken in the UNKE group. Patients in this group used three or more ASDs in 40.9% of the cases, compared to the KE group, 25.0%. However, other causes cannot be excluded.

Lastly, we would like to emphasize the importance of our findings concerning the latest ILAE Classification of the Epilepsies [61]. It suggests a division into three categories: focal epilepsy, generalized epilepsy, or epileptic encephalopathy, and development encephalopathy. It is well-known that patients with encephalopathies can suffer from both focal and generalized seizures. In this respect, we obtained the following KD effectiveness groups: focal seizures only (25.0%), tonic-clonic seizures only (40.0%), simultaneous focal and tonic-clonic seizures (52.9%). This suggests that the identification of focal seizures does not necessarily imply a lower efficacy of KD. On the contrary, provided it is not the only form of episode, this suggests the possibility of the KD’s higher efficacy. Additionally, these results exemplify the practical usefulness of the new proposed classification in identifying patients who might benefit from KD as a form of treatment.

## 5. Conclusions

Based on obtained data, it can be concluded that treatment with the KD is a safe and effective form of epilepsy therapy. Because of the KD, the overall usage of ASDs was reduced. The KD was the least effective in reducing focal seizures in both known and unknown etiology groups. The most significant benefit from this form of treatment can be expected in patients who have both focal and generalized seizures, that is, in a group of patients with developmental and epileptic encephalopathies of known molecular or cytogenetic background. Finding the occurrence of focal seizures in a patient with epilepsy does not exclude this form of anti-seizure treatment. The new proposed ILAE Classification and Definition of Epilepsy Syndromes can significantly help estimate the potential benefit of dietary treatment and allow earlier use of this form of therapy. Considering the effectiveness and minor side effects of dietary treatment, it seems reasonable to recommend this form of treatment in all patients with epilepsy. The search for the genetic basis of epilepsy seems reasonable in all patients, both in patients with generalized and focal seizures, in the case of epilepsy or epileptic encephalopathy.

## Figures and Tables

**Table 1:** General characteristic of the cohort.

	Determined Etiology	Undetermined Etiology	All Cases
Cases (n, %)	20, 47.62	22, 52.38	42, 100.00
Gender (n, %)	F (11, 47.82), M (9, 47.36)	F (12, 52.17), M (10, 52.63)	F (23, 54.76), M (19, 45.24)
Mean age ^†^ (years, range)	10.35, 3.00–18.58	7.72, 2.00–14.50	8.97, 2.00–18.58
Mean time of treatment duration (years, range)	2.30, 0.17–5.00	2.25, 0.33–5.00	2.28, 0.17–5.00
Mean age of the treatment initiation (years, range)	8.05, 1.00–18.00	5.47, 0.50–12.50	6.70, 0.50–18.00
Mean age of first seizure (years, range)	2.19, 0.00–12.00	1.69, 0.17–6.00	1.92, 0.00–12.00

F—female; M—male; ^†^—at the time of assessment.

**Table 2:** Efficacy of the ketogenic diet treatment depending on the recognized genetic background or symptomatic diagnosis.

Seizures Reduction	100%		>90%		75–90%		51–75%		≤50%	
KE	N	Genetic Background	N	Genetic Background	N	Genetic Background	N	Genetic Background	N	Genetic Background
	9	*DEPDC5*—3 *SLC2A1*—5 *SYNGAP1*—1	1	trisomy 21—1	6	*CACNA1S*—1 *CHD2*—1 *SCN1A*—1 *SCN8A*—1 *ZEB2*—1 del15q11-13—1	2	*PIGN*—1 *SCN1A*—1	2	*KIF1A*—1 *SCN8A*—1
**%**	**45.00**		**5.00**		**30.00**		**10.00**		**10.00**	
**UNKE**	**N**	**Diagnosis**	**N**	**Diagnosis**	**N**	**Diagnosis**	**N**	**Diagnosis**	**N**	**Diagnosis**
	3	DS.—2 JAE—1	4	AS-like—1 DS.—1 LGS—1 RS-like—1	2	AS-like—1 RS-like—1	2	DS.—1 FIRES—1	11	DS.—1 UNFE—7 LGS—2 RS-like—1
%	13.63		18.18		9.09		9.09		50.00	

The seizure reduction percentage considered as effective treatment is marked in gray. N—number of cases; KE—determined etiology; UNKE—undetermined etiology.

**Table 3:** The level of epileptic seizure reduction achieved after ketogenic diet therapy depending on the type of seizure.

	Seizure Reduction		
	>75%	>50%	≤50%
Seizure Type			
Clonic	100.0% (1/1)	100.0% (1/1)	0.0% (0/1)
Myoclonic	73.7% (14/19)	89.5% (17/19)	10.5% (2/19)
Absence	78.9% (15/19)	84.2% (16/19)	15.8% (3/19)
Atonic	75.0% (9/12)	83.3% 10/12	16.7% (2/12)
Tonic-clonic	57.7% (15/26)	65.4% (17/26)	34.6% (9/26)
Tonic	60.0% (3/5)	60.0% (3/5)	40.0% (2/5)
Partial	38.1% (8/21)	42.9% (9/21)	57.1% (12/21)
Non-partial	76.2% (16/21)	90.5% (19/21)	9.5% (2/21)
Non-tonic-clonic	62.5% (10/16)	75.0% (12/16)	25.0% (4/16)
Partial and generalized	47.1% (8/17)	52.9% (9/17)	47.1% (8/17)
Tonic-clonic only	30.0% (3/10)	40.0% (4/10)	60.0% (6/10)
Partial only	25.0% (1/4)	25.0% (1/4)	75.0% (3/4)

**Table 4:** The type of ketogenic diet treatment used in patients.

Diet Type	KE	UNKE	Effective ^†^	Ineffective	Total
1:1 ^‡^	2	0	1	1	2
1.5:1	1	2	2	1	3
2:1	3	4	5	2	7
3:1	5	9	8	6	14
3.5:1	1	0	1	0	1
4:1	1	0	1	0	1
MAD	6	7	9	4	13
LGIT	1	0	1	0	1
	**KE Non-Partial Seizures**	**Effective ^†^**	**UNKE Non-Partial Seizures**	**Effective ^†^**	**Total**
3:1	3	3	3	3	6
MAD	4	4	1	1	5
	**KE with Partial Seizures**	**Effective ^†^**	**UNKE with Partial Seizures**	**Effective ^†^**	**Total**
3:1	2	2	6	1	8
MAD	2	1	6	3	8

KE—seizures of known etiology; UNKE—seizures of unknown etiology; ^†^—diet was found to be effective if seizure reduction was >50%; ^‡^—the ratio of fats to proteins and carbohydrates; MAD—modified Atkins diet; LGIT—low-glycemic index treatment.

**Table 5:** Presentation of most commonly used anti-seizure drugs and number of them in specific groups.

	LEV	VPA	LEV + VPA	ETS	
Cases	18	15	7	10	
Effective	72.2% (13/18)	66.7% (10/15)	71.4% (5/7)	90.0% (9/10)	
Ineffective	27.8% (5/18)	33.3% (5/15)	28.6% (2/7)	10.0% (1/10)	
Effective KE	100% (9/9)	100% (5/5)	100% (3/3)	100% (5/5)	
Ineffective KE	0.0% (0/9)	0.0% (0/5)	0.0% (0/3)	0.0% (0/5)	
Effective UNKE	44.4% (4/9)	50.0% (5/10)	50.0% (2/4)	80.0% (4/5)	
Ineffective UNKE	55.6% (5/9)	50.0% (5/10)	50.0% (2/4)	20.0% (1/5)	
Effective Partial	50.0% (5/10)	28.6% (2/7)	33.3% (1/3)	66.7% (2/3)	
Ineffective Partial	50.0% (5/10)	71.4% (5/7)	66.7% (2/3)	33.3% (1/3)	
Effective GTCS	75.0% (9/12)	50.0% (4/8)	75.0% (3/4)	100% (5/5)	
Ineffective GTCS	25.0% (3/12)	50.0% (4/8)	25.0% (1/4)	0.0% (0/5)	
	**Overall**	**KE**	**UNKE**	**Partial**	**Non-Partial**
0 ASD	9.5% (4/42)	20.0% (4/20)	0.0% (0/22)	4.8% (1/21)	14.3% (3/21)
1 ASD	26.2% (11/42)	30.0% (6/20)	22.7% (5/22)	23.8% (5/21)	28.6% (6/21)
2 ASDs	31.0% (13/42)	20.0% (4/20)	40.9% (9/22)	23.8% (5/21)	38.1% (8/21)
3 ASDs	23.8% (10/42)	20.0% (4/20)	27.3% (6/22)	33.3% (7/21)	14.3% (3/21)
4 ASDs	9.5% (4/42)	5.0% (1/20)	13.6% (3/22)	14.3% (3/21)	4.8% (1/21)
≥2 ASDs	64.3% (27/42)	45.0% (9/20)	81.8% (18/22)	71.4% (15/21)	57.1% (12/21)
≥3 ASDs	33.3% (14/42)	25.0% (5/20)	40.9% (9/22)	47.6% (10/21)	19.1% (4/21)
Discontinuation of ≥1 ASD during the KD treatment	38.1% (16/42)	40.0% (8/20)	36.4% (8/22)	33.3% (7/21)	42.9% (9/21)
Addition of ≥1 ASD during the KD treatment	40.5% (17/42)	25.0% (5/20)	54.5% (12/22)	47.6% (10/21)	33.3% (7/21)

LEV—levetiracetam; VPA—valproic acid; ETS—ethosuximide; ASD—anti-seizure drug; KE—seizures of known etiology; UNKE—seizures of unknown etiology; Partial—occurrence of partial seizures; Non-partial—partial seizures were not observed; KD—the ketogenic diet; GTCS—generalized tonic-clonic seizures.

## Data Availability

The results can be found in the medical histories of the patients archived at the Chair and Department of Developmental Neurology, University of Medical Sciences Poznan, Poland.

## References

[1] Wilder R.M. (1921). The effect on ketonemia on the course of epilepsy. Mayo Clin. Bull..

[2] Pulford D.S. (1927). Ketogenic Diets for Epileptics. Calif. West Med..

[3] Viggiano A., Stoddard M., Pisano S., Operto F.F., Iovane V., Monda M., Coppola G. (2016). Ketogenic diet prevents neuronal firing increase within the substantia nigra during pentylenetetrazole-induced seizure in rats. Brain Res. Bull..

[4] Zhang Y., Xu J., Zhang K., Yang W., Li B. (2017). The Anticonvulsant Effects of Ketogenic Diet on Epileptic Seizures and Potential Mechanisms. Curr. Neuropharmacol..

[5] Lefevre F., Aronson N. (2000). Ketogenic diet for the treatment of refractory epilepsy in children: A systematic review of efficacy. Pediatrics.

[6] Kinsman S.L., Vining E.P.G., Quaskey S.A., Mellits D., Freeman J.M. (1992). Efficacy of the ketogenic diet for intractable seizure disorders: Review of 58 cases. Epilepsia.

[7] Falsaperla R., D’Angelo G., Praticò A.D., Mauceri L., Barbagallo M., Pavone P., Catanzaro S., Gitto E., Corsello G., Ruggieri M. (2020). Ketogenic diet for infants with epilepsy: A literature review. Epilepsy Behav..

[8] Schoeler N.E., Leu C., Balestrini S., Mudge J.M., Steward C.A., Frankish A., Leung M.-A., Mackay M., Scheffer I., Williams R. (2018). Genome-wide association study: Exploring the genetic basis for responsiveness to ketogenic dietary therapies for drug-resistant epilepsy. Epilepsia.

[9] Verrotti A., Iapadre G., Di Francesco L., Zagaroli L., Farello G. (2020). Diet in the Treatment of Epilepsy: What We Know So Far. Nutrients.

[10] Wheless J.W. (2008). History of the ketogenic diet. Epilepsia.

[11] Barzegar M., Afghan M., Tarmahi V., Behtari M., Khamaneh S.R., Raeisi S. (2019). Ketogenic diet: Overview, types, and possible anti-seizure mechanisms. Nutr. Neurosci..

[12] Bough K.J., Rho J.M. (2007). Anticonvulsant Mechanisms of the Ketogenic Diet. Epilepsia.

[13] Maalouf M., Rho J.M., Mattson M.P. (2009). The neuroprotective properties of calorie restriction, the ketogenic diet, and ketone bodies. Brain Res. Rev..

[14] Martin K., Jackson C.F., Levy R.G., Cooper P.N. (2016). Ketogenic diet and other dietary treatments for epilepsy. Cochrane Database Syst. Rev..

[15] Brouns F. (2018). Overweight and diabetes prevention: Is a low-carbohydrate–high-fat diet recommendable?. Eur. J. Nutr..

[16] Kossoff E.H., Krauss G.L., McGrogan J.R., Freeman J.M. (2003). Efficacy of the Atkins diet as therapy for intractable epilepsy. Neurology.

[17] Kossoff E.H., Dorward J.L. (2008). The modified Atkins diet. Epilepsia.

[18] Kossoff E.H., Dorward J.L., Molinero M.R., Holden K.R. (2008). The modified Atkins diet: A potential treatment for developing countries. Epilepsia.

[19] Pfeifer H.H., Thiele E.A. (2005). Low-glycemic-index treatment: A liberalized ketogenic diet for treatment of intractable epilepsy. Neurology.

[20] Huttenlocher P.R., Wilbourn A.J., Signore J.M. (1971). Medium-chain triglycerides as a therapy for intractable childhood epilepsy. Neurology.

[21] Watanabe M., Tuccinardi D., Ernesti I., Basciani S., Mariani S., Genco A., Manfrini S., Lubrano C., Gnessi L. (2020). Scientific evidence underlying contraindications to the ketogenic diet: An update. Obes. Rev..

[22] Watanabe M., Tozzi R., Risi R., Tuccinardi D., Mariani S., Basciani S., Spera G., Lubrano C., Gnessi L. (2020). Beneficial effects of the ketogenic diet on nonalcoholic fatty liver disease: A comprehensive review of the literature. Obes. Rev..

[23] Bruci A., Tuccinardi D., Tozzi R., Balena A., Santucci S., Frontani R., Mariani S., Basciani S., Spera G., Gnessi L. (2020). Very Low-Calorie Ketogenic Diet: A Safe and Effective Tool for Weight Loss in Patients with Obesity and Mild Kidney Failure. Nutrients.

[24] Van der Louw E.J., Williams T.J., Henry-Barron B.J., Olieman J.F., Duvekot J.J., Vermeulen M.J., Bannink N., Williams M., Neuteboom R.F., Kossoff E.H. (2016). Ketogenic diet therapy for epilepsy during pregnancy: A case series. Seizure.

[25] Ye F., Li X.-J., Jiang W.-L., Sun H.-B., Liu J. (2015). Efficacy of and Patient Compliance with a Ketogenic Diet in Adults with Intractable Epilepsy: A Meta-Analysis. J. Clin. Neurol..

[26] Neal E.G., Chaffe H., Schwartz R.H., Lawson M.S., Edwards N., Fitzsimmons G., Whitney A., Helen Cross J. (2009). A randomized trial of classical and medium-chain triglyceride ketogenic diets in the treatment of childhood epilepsy. Epilepsia.

[27] Miranda M.J., Mortensen M., Povlsen J.H., Nielsen H., Beniczky S. (2011). Danish study of a modified Atkins diet for medically intractable epilepsy in children: Can we achieve the same results as with the classical ketogenic diet?. Seizure.

[28] Kim J.A., Yoon J.R., Lee E.J., Lee J.S., Kim J.T., Kim H.D., Kang H.C. (2016). Efficacy of the classic ketogenic and the modified Atkins diets in refractory childhood epilepsy. Epilepsia.

[29] Wijnen B.F., de Kinderen R.J., Lambrechts D.A., Postulart D., Aldenkamp A.P., Majoie M.H., Evers S.M. (2017). Long-term clinical outcomes and economic evaluation of the ketogenic diet versus care as usual in children and adolescents with intractable epilepsy. Epilepsy Res..

[30] Freitas L.L., Soriano E., Muccioli C., Hofling-Lima A.L., Belfort R. (2007). Efficacy and tolerability of a combined moxifloxacin/dexamethasone formulation for topical prophylaxis and reduction of inflammation in phacoemulsification: A comparative, double masked clinical trial. Curr. Med. Res. Opin..

[31] Zarnowska I.M. (2020). Therapeutic Use of the Ketogenic Diet in Refractory Epilepsy: What We Know and What Still Needs to Be Learned. Nutrients.

[32] Kraeuter A.-K., Guest P.C., Sarnyai Z. (2019). The Therapeutic Potential of Ketogenic Diet Throughout Life: Focus on Metabolic. Neurodev. Neurodegener. Disord..

[33] Than K.D., Kossoff E.H., Rubenstein J.E., Pyzik P.L., McGrogan J.R., Vining E.P.G. (2005). Can You Predict an Immediate, Complete, and Sustained Response to the Ketogenic Diet?. Epilepsia.

[34] Freitas A., Da Paz J.A., Casella E.B., Marques-Dias M.J. (2007). Ketogenic diet for the treatment of refractory epilepsy: A 10 year experience in children. Arq. Neuro-Psiquiatr..

[35] Vining E.P.G., Freeman J.M., Ballaban-Gil K., Camfield C.S., Camfield P.R., Holmes G.L., Shinnar S., Shuman R., Trevathan E., Wheless J.W. (1998). A Multicenter Study of the Efficacy of the Ketogenic Diet. Arch. Neurol..

[36] Martin-McGill K.J., Bresnahan R., Levy R.G., Cooper P.N. (2020). Ketogenic diets for drug-resistant epilepsy. Cochrane Database Syst. Rev..

[37] Kass H.R., Winesett S.P., Bessone S.K., Turner Z., Kossoff E.H. (2016). Use of dietary therapies amongst patients with GLUT1 deficiency syndrome. Seizure.

[38] Herrero J.R., Villarroya E.C., Gutiérrez-Solana L.G., Alcolea B.G., Fernández B.G., Macfarland L.P., Pedrón-Giner C. (2021). Classic Ketogenic Diet and Modified Atkins Diet in SLC2A1 Positive and Negative Patients with Suspected GLUT1 Deficiency Syndrome: A Single Center Analysis of 18 Cases. Nutrients.

[39] Elamin M., Ruskin D.N., Sacchetti P., Masino S.A. (2020). A unifying mechanism of ketogenic diet action: The multiple roles of nicotinamide adenine dinucleotide. Epilepsy Res..

[40] Cervenka M.C., Henry B.J., Felton E.A., Patton K., Kossoff E.H. (2016). Establishing an Adult Epilepsy Diet Center: Experience, efficacy and challenges. Epilepsy Behav..

[41] Freeman J.M., Vining E.P., Pillas D.J., Pyzik P.L., Casey J.C., Millicent T. (1998). The efficacy of the ketogenic diet—1998: A prospective Evaluation of Intervention in 150 Children. Pediatrics.

[42] Liu X., Chen J., Zhu M., Zheng G., Guo H., Lu X., Wang X., Yang X. (2019). Three and Six Months of Ketogenic Diet for Intractable Childhood Epilepsy: A Systematic Review and Meta-Analysis. Front. Neurol..

[43] Yan N., Xin-Hua W., Lin-Mei Z., Yi-Ming C., Wen-Hui L., Yuan-Feng Z., Shui-Zhen Z. (2018). Prospective study of the efficacy of a ketogenic diet in 20 patients with Dravet syndrome. Seizure.

[44] Ko A., Jung D.E., Kim S.H., Kang H.-C., Lee J.S., Lee S.-T., Choi J.R., Kim H.D. (2018). The Efficacy of Ketogenic Diet for Specific Genetic Mutation in Developmental and Epileptic Encephalopathy. Front. Neurol..

[45] Tsai M.-H., Chan C.-K., Chang Y.-C., Yu Y.-T., Chuang S.-T., Fan W.-L., Li S.-C., Fu T.-Y., Chang W.-N., Liou C.-W. (2017). DEPDC5 mutations in familial and sporadic focal epilepsy. Clin. Genet..

[46] Bisulli F., Licchetta L., Baldassari S., Pippucci T., Tinuper P. (2016). DEPDC5 mutations in epilepsy with auditory features. Epilepsia.

[47] Mignot C., von Stülpnagel C., Nava C., Ville D., Sanlaville D., Lesca G., Rastetter A., Gachet B., Marie Y., Korenke G.C. (2016). Genetic and neurodevelopmental spectrum ofSYNGAP1-associated intellectual disability and epilepsy. J. Med. Genet..

[48] Agarwal M., Johnston M.V., Stafstrom C.E. (2019). SYNGAP1 mutations: Clinical, genetic, and pathophysiological features. Int. J. Dev. Neurosci..

[49] Nicita F., Ginevrino M., Travaglini L., D’Arrigo S., Zorzi G., Borgatti R., Terrone G., Catteruccia M., Vasco G., Brankovic V. (2020). Heterozygous KIF1A variants underlie a wide spectrum of neurodevelopmental and neurodegenerative disorders. J. Med. Genet..

[50] Gardella E., Marini C., Trivisano M., Fitzgerald M.P., Alber M., Howell K.B., Darra F., Siliquini S., Bölsterli B.K., Masnada S. (2018). The phenotype of SCN8A developmental and epileptic encephalopathy. Neurology.

[51] Kossoff E.H., Zupec-Kania B.A., Auvin S., Ballaban-Gil K.R., Christina Bergqvist A.G., Blackford R., Buchhalter J.R., Caraballo R.H., Helen Cross J., Dahlin M.G. (2018). Optimal clinical management of children receiving dietary therapies for epilepsy: Updated recommendations of the International Ketogenic Diet Study Group. Epilepsia Open.

[52] Caraballo R., Vaccarezza M., Cersósimo R., Rios V., Soraru A., Arroyo H., Agosta G., Escobal N., Demartini M., Maxit C. (2011). Long-term follow-up of the ketogenic diet for refractory epilepsy: Multicenter argentinean experience in 216 pediatric patients. Seizure.

[53] Nei M., Ngo L., Sirven J.I., Sperling M.R. (2014). Ketogenic diet in adolescents and adults with epilepsy. Seizure.

[54] Caraballo R.H. (2011). Nonpharmacologic treatments of Dravet syndrome: Focus on the ketogenic diet. Epilepsia.

[55] Dahlin M.G., Beck O.M., Åmark P.E. (2006). Plasma Levels of Antiepileptic Drugs in Children on the ketogenic diet. Pediatr. Neurol..

[56] Coppola G., Verrotti A., D’Aniello A., Arcieri S., Operto F.F., Della Corte R., Ammendola E., Pascotto A. (2010). Valproic acid and phenobarbital blood levels during the first month of treatment with the ketogenic diet. Acta Neurol. Scand..

[57] Van der Louw E.J., Desadien R., Vehmeijer F., van der Sijs H., Catsman-Berrevoets C.E., Neuteboom R.F. (2015). Concomitant lamotrigine use is associated with decreased efficacy of the ketogenic diet in childhood refractory epilepsy. Seizure.

[58] Schwartz R.H., Eaton J., Bower B.D., Aynsley-Green A. (1989). Ketogenic diets in the treatment of epilepsy: Short-term clinical effects. Dev. Med. Child Neurol..

[59] El-Rashidy O.F., Nassar M.F., Abdel-Hamid I.A., Shatla R.H., Abdel-Hamid M.H., Gabr S.S., Mohamed S.G., El-Sayed W.S., Shaaban S.Y. (2013). Modified Atkins diet vs classic ketogenic formula in intractable epilepsy. Acta Neurol. Scand..

[60] Poorshiri B., Barzegar M., Tahmasebi S., Shiva S., Raeisi S., Ebadi Z. (2019). The efficacy comparison of classic ketogenic diet and modified Atkins diet in children with refractory epilepsy: A clinical trial. Acta Neurol. Belg..

[61] Proposed Classification: Syndromes in Children. https://www.ilae.org/guidelines/definition-and-classification/proposed-classification-and-definition-of-epilepsy-syndromes/proposed-classification-syndromes-in-children.

